# Role of interleukin-4 in pathogenesis of oral lichen planus: A systematic review

**DOI:** 10.4317/medoral.23460

**Published:** 2020-03-06

**Authors:** Solmaz Pourzare Mehrbani, Paria Motahari, Fatemeh Pournagi Azar, Morteza Akbarzadeh Ahari

**Affiliations:** 1 Assistant Professor, Department of Oral Medicine, Faculty of Dentistry, Tabriz University of Medical Sciences, Tabriz, Iran; 2 Assistant Professor, Department of restorative dentistry, Faculty of Dentistry, Tabriz University of Medical Sciences, Tabriz, Iran; 3 Department of Oral Medicine, Faculty of Dentistry, Tabriz University of Medical Sciences, Tabriz, Iran

## Abstract

**Background:**

Oral lichen planus (OLP) is a premalignant mucocutaneous disease that affects 1-2% of the adult population. Immunological factor may act as etiological factor. The cellular immune cells such as T cells are important in pathogenesis of OLP. Interleukin-4 (IL-4) is secreted by T-helper 2 (Th2). Several studies have been carried out on the role of IL-4 in OLP. The aim of this study was to review the level of IL-4 in OLP, effective factors in the production of IL-4 and its role in the development of OLP.

**Material and Methods:**

A search in PubMed was performed on the literature published from 2000 until august 2019 using the following keywords: “oral lichen planus” or “OLP” and “interleukin-4” or “IL-4”.

**Results:**

Originally, 37 articles were considered, of which 28 case-control articles were selected according to the inclusion/exclusion criteria.

**Conclusions:**

This review study shows that IL-4 plays a key role in the development of OLP. According to the past studies, there are several factors contributing to the production of this cytokine. Identification of the routes of production of IL-4 and its role in OLP might be useful for development of new preventive and therapeutic methods in management of patients with OLP.

** Key words:**Interleukin 4, oral lichen planus, pathogenesis.

## Introduction

Oral lichen planus (OLP) is recognized as a chronic inflammatory disease with other systemic disorders. Its prevalence ranges from 1% to 2% among the general population. It often involves middle-aged patients and is more common in women than in men (1,2). Although the etiology of OLP is unknown, various mechanisms have been suggested to be involved in its pathogenesis (specific immune response to antigens, immune response, humoral immunity, and nonspecific mechanisms). OLP is a T-cell-induced autoimmune disorder in which CD8 + cells can induce apoptosis in oral epithelial cells. T helper cells (Th) are traditionally divided into two subgroups (Th1 and Th2). Accordingly, impaired immune regulation has been implicated in the cause of OLP. Cytokines can play a mediating role between keratinocytes and inflammatory cells, and they also play important roles in damaging keratinocytes (3-6). Interleukin-4 (IL-4) is an important cytokine that is responsible for the secretion of other cytokines. It plays a central role in regulating antibody production and humoral immune response to differentiate Th2 cells (7-10). Several studies have been carried out on the role of IL-4 in OLP. Some studies have examined IL-4 levels in the tissues; saliva and serum in OLP, and contradictory results have been presented. Other studies have examined the factors affecting the production of IL-4 and the mechanisms of lesions of OLP formation by this cytokine. Our purpose in this study has been placed on the levels of IL-4 in different samples, production of IL-4 and its role in the development of OLP in this review. Identification of the routes of production of IL-4 and its role in OLP might be useful for development of new preventive and therapeutic methods in management of patients with OLP as well.

## Material and Methods

This systematic review was conducted based on the Preferred Reporting Items for Systematic reviews and Meta-Analyses (PRISMA) statement for reporting systematic reviews (11).

- Search strategies

The PubMed (MEDLINE) database of the United States MEDLINE was used as search source. The keywords were selected based on Medical Subject Heading (MeSH) terms. The studies were retrieved by searching the key terms “oral lichen planus” or “OLP” and “interleukin-4” or “IL-4” in PubMed databases from 2000 up to august 2019.

- Study selection and selection criteria

A protocol was used for establishment of the inclusion and exclusion criteria. The studies on the association between OLP and IL-4 were selected without restrictions of variants of OLP. To select the studies, all obtained English language reports were reviewed, and titles and abstracts were screened for relevance. Studies were excluded if they were review or animal studies. Duplicate publications (risk of bias), were removed from the study.

- Data extraction

The studies were checked by one author (P.M.) to extract all the relevant data. Another author (M.A) reevaluated the data. Disagreements were resolved with the third author’s discussion (S.P.M).

## Results

- Study selection

**Figure 1:**
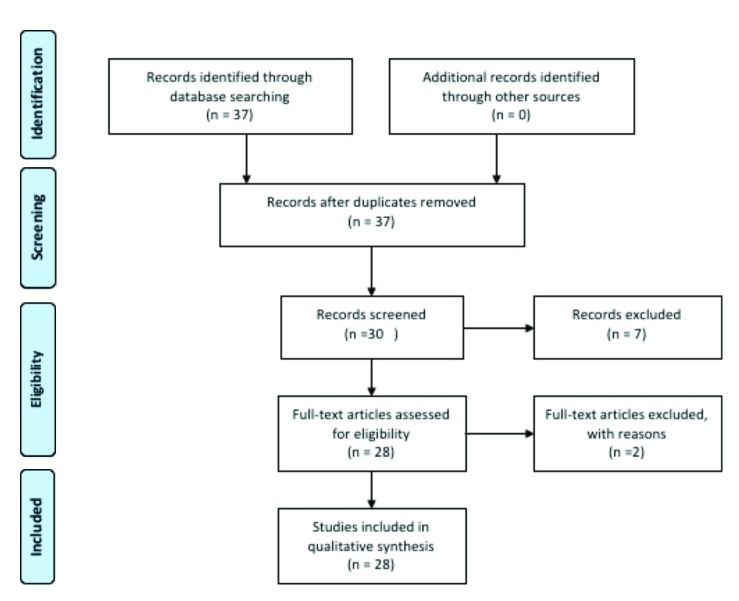
The PRISMA flowchart of the study selection.

In an initial research, 37 articles were identified through electronic database. The full texts of these studies were assessed for eligibility and 9 studies were excluded with reasons (5 studies were review/systematic review, 1 reported the polymorphisms of other cytokine, 1 was performed on animals, 1 was performed on cutaneous lichen planus and 1 study was published in Chinese) that shown in Fig. 1.

Finally, 28 studies were evaluated. Of the 28 articles evaluated, 14 articles evaluated the IL-4 levels in the different samples, which are detailed in Table 1 (12-25); nine articles evaluated the effect of different factors on the production of IL-4 that also shown in Table 2 (26-34). One article examined the polymorphism of the IL-4 gene in patient with lichen planus, one article about the amount of IL-4 producing cells; one article examined the effect of IL-4 on CD 275+ cells and one article compared IL-4+ T cells levels between OLP and SCC specimen. The details of these five studies are shown in the Table 3 (35-39).

**Table 1:**
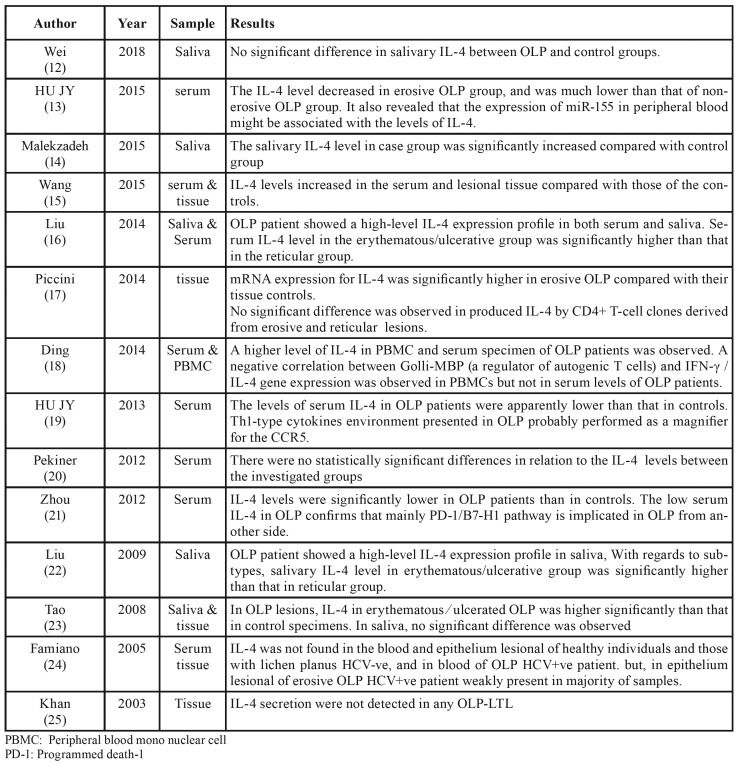
Articles evaluated the IL-4 levels in the different samples.

**Table 2:**
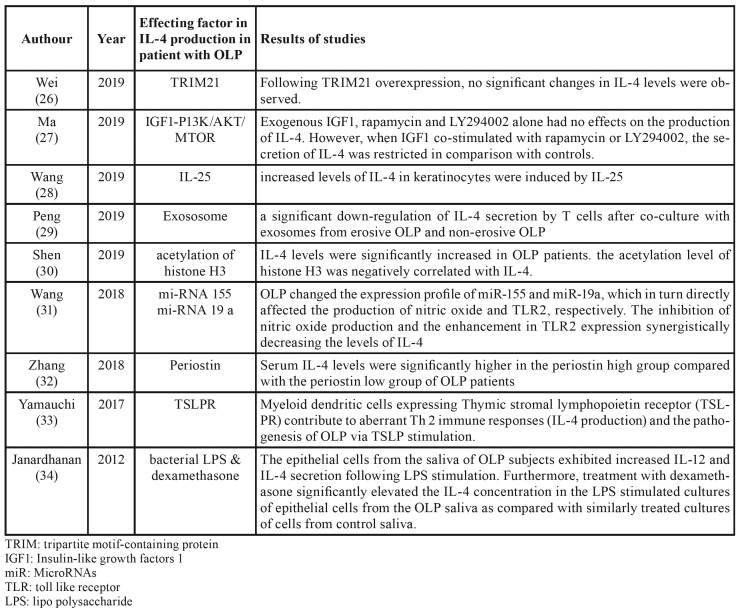
Articles evaluated the effect of different factors on the production of IL-4.

**Table 3:**
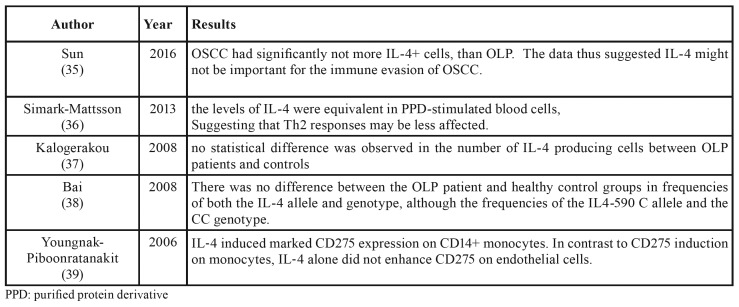
Other lichen planus article.

## Discussion

Although the exact cause of OLP is still unknown, it has been widely accepted that immunodeficiency cases are very important. The role of Th1 / Th2 imbalance in the pathogenesis of OLP has been widely studied among several etiologic agents in the Past years (4,7,8). Given the expression of Th1 cytokines by T cells in OLP lesion lymphocytes and the pathological hypothesis of OLP that Th1-activated auto cytotoxic CD8 T cells induce apoptosis of keratinocytes, Sugerman *et al*. suggested that OLP was characterized by Th1 cytokines (10). However, concomitant expression of Th1 and Th2 cytokines is observed in localized OLP lesions and tissue secretions. In addition, saliva, serum, and peripheral blood mononuclear cells from OLP patients have a complex expression profile of Th1 and Th2 cytokines. There is no doubt that both Th1 and Th2 immune responses are involved in the development of OLP. Interferon gamma (IFN-γ) and IL-4, the most characteristic of Th1 and Th2 cytokines, regulate T cell differentiation and Th1 / Th2 balance, respectively, in physiological and pathological immune processes. IFN-γ is involved in the maturation and activation of cytotoxic CD8 T cells and maintenance of the expression of major class II tissue adhesion molecules, thus participating in keratinocyte apoptosis and chronic OLP. IL-4 disease, on the other hand, for Th2 cell differentiation and it has an important role in regulating antibody production and humoral immune response (4,7,8).

Table 1 presents contradictory results on the levels of IL-4 in different samples. This disagreement may be due in part to patient differences, subgroups of OLP, research methods, or sample size, but also suggests that mechanisms such as the role of T regulatory cells and new Th cell subsets may exist, in the etiology of OLP except for the Th1 / Th2 imbalance. Another important factor to consider is the interaction of genes and cytokines as well as the effect of gene polymorphisms on cytokine production. Indeed, a study of Chinese ethnicity showed that IL-4 gene polymorphism had a positive effect on OLP sensitivity and prognosis (38). It may therefore explain the differences between results in numerous studies. In addition, according to the studies in the present review, none of them examined the effect of age and sex on IL-4 levels, although we believe that these factors should be taken into account in future studies. The most important point to be drawn from these Tables is that the levels of interleukin but not its secretory cells are higher in most studies in the Lichen Planus group than in healthy individuals, thus indicating that IL-4 may represent a potential salivary biomarker for the disease. Taking advantage of easy access and noninvasive collection by intermediate-educated individuals, whole saliva offers a cost-effective way to monitor recurrent diseases and screen large populations in studies of Systemic diseases, especially oral diseases, which is consistent with a recent systematic review (40). In most of these studies, interleukin-4 levels are higher in the erosive form than in the reticular type, which may be attributed to more inflammation and infection in the erosive form, or to the possible role of interleukin in the conversion of the reticular to the erosive type through impaired keratinocyte repair. Another point to note is that the levels of interleukin-4 in HCV-ve are not different from those of HCV+ve, so this cytokine is not an important factor in the development of lichen planus in hepatitis c-positive individuals (24). Also, the level of this cytokine in patients with SCC is lower than in those with lichen planus. It can be concluded that interleukin is not an important factor in the development of malignancy in lichen planus (35).

Youngnak-*Pi*boonratanakit *et al* showed that IL-4 induced CD275 expression on monocytes, that is more important for Th2 differentiation and effector functions then for Th1 responses.

This review study shows that IL-4 plays a key role in the development of OLP. According to the past studies, there are several factors contributing to the production of this cytokine. In addition, IL-4 causes OLP lesions through its effect on various cells. It is hoped that this review article raises our awareness about the role of IL-4 in the etiopathogenesis of lichen planus and open up new ways of preventing and treating.
